# An Effective Camera-to-Lidar Spatiotemporal Calibration Based on a Simple Calibration Target

**DOI:** 10.3390/s22155576

**Published:** 2022-07-26

**Authors:** Lazaros Grammatikopoulos, Anastasios Papanagnou, Antonios Venianakis, Ilias Kalisperakis, Christos Stentoumis

**Affiliations:** 1 up2metric P.C., Engineering-Research-Software Development, Michail Mela 21, 11521 Athens, Greece; anastasis.papanagnou@up2metric.com (A.P.); antonis.venianakis@up2metric.com (A.V.); ilias@up2metric.com (I.K.); christos@up2metric.com (C.S.); 2 Department of Surveying and Geoinformatics Engineering, University of West Attica, Ag. Spyridonos Str., Egaleo, 12243 Athens, Greece

**Keywords:** extrinsic calibration, Lidar, camera, calibration target, temporal calibration

## Abstract

In this contribution, we present a simple and intuitive approach for estimating the exterior (geometrical) calibration of a Lidar instrument with respect to a camera as well as their synchronization shifting (temporal calibration) during data acquisition. For the geometrical calibration, the 3D rigid transformation of the camera system was estimated with respect to the Lidar frame on the basis of the establishment of 2D to 3D point correspondences. The 2D points were automatically extracted on images by exploiting an AprilTag fiducial marker, while the detection of the corresponding Lidar points was carried out by estimating the center of a custom-made retroreflective target. Both AprilTag and Lidar reflective targets were attached to a planar board (calibration object) following an easy-to-implement set-up, which yielded high accuracy in the determination of the center of the calibration target. After the geometrical calibration procedure, the temporal calibration was carried out by matching the position of the AprilTag to the corresponding Lidar target (after being projected onto the image frame), during the recording of a steadily moving calibration target. Our calibration framework was given as an open-source software implemented in the ROS platform. We have applied our method to the calibration of a four-camera mobile mapping system (MMS) with respect to an integrated Velodyne Lidar sensor and evaluated it against a state-of-the-art chessboard-based method. Although our method was a single-camera-to-Lidar calibration approach, the consecutive calibration of all four cameras with respect to the Lidar sensor yielded highly accurate results, which were exploited in a multi-camera texturing scheme of city point clouds.

## 1. Introduction

In recent years, there has been an increasing demand for efficient data utilization derived from various sources. The fusion of 3D range data acquired from laser scanners with color information from RGB cameras is a typical example of multimodal data exploitation, commonly adopted in 3D city modeling scenarios. Several mobile mapping systems (MMSs) have been reported and used [1,2,3], which consist primarily of multiple RGB cameras (multi-camera rig) along with a Lidar sensor mounted on top of a moving vehicle/robot.

An indispensable procedure during multimodal data elaboration is the accurate recovery of the sensor’s relative orientation (rotation and translation), a process also known as external calibration [4,5,6,7]. Depending on the characteristics of the specific methodology employed for 3D reconstruction (Lidar-based SLAM, visual SLAM of SFM), the calibration approach can significantly affect the achieved geometrical accuracy as well as the visual quality of the derived 3D maps (global point clouds), mostly due to severe data misalignments. Such calibration techniques have been widely studied in the Computer Vision and Robotics literature, while quite a few of them have led to the development of corresponding software applications that enable the retrieval of the extrinsic and/or intrinsic sensor parameters following some simple steps.

Among methods that utilize special targets for Lidar–camera calibration [8,9,10,11,12], the most widespread category, indisputably, comprises the chessboard-based approaches. This is because of the flexibility of the specific calibration object—in general terms, a chessboard can be easily extracted from image and range data—and the ability it provides for simultaneous estimation of the intrinsic camera parameters. In the preliminary approach of Unnikrishnan et al. [4], chessboard correspondences among image and range data must be selected by the user in an interactive GUI. In the same fashion, Scaramuzza et al. [5] described a calibration toolbox in which point correspondences could be established manually. Their approach does not require any calibration objects.

Having fixed the interior orientation of the camera, the method of Zhang et al. [6] was the first to identify the rigid transformation between a camera and a 2D laser range finder from sufficient poses of a planar chessboard. For each pose of the chessboard pattern, the detected Lidar points on the board and the inferred camera pose with respect to the chessboard imposed linear constraints to the camera’s position and rotation relative to the laser range finder. The closed-form solution was further refined by nonlinear minimization (instead of the previous algebraic); optionally, it may also optimize the camera’s internal parameters through global adjustment. This calibration tool is available from the authors on request.

Padley et al. [7] exploited the constraint provided by the laser points of the planar surface and the normal of the plane detected from the image data in order to estimate the rigid body transformation between the two sensors. A minimum of three non-coplanar views of the chessboard were required to estimate the extrinsic calibration parameters. A modification of Padley’s work was recently proposed [13]; it consecutively estimated the rotation and the translation component of the rigid transformation. In the final step, the extrinsic parameters were optimized by an iterative minimization scheme.

In a similar manner, Zou et al. [14] recently reduced the minimum number of chessboard poses required to externally calibrate a 3D laser scanner and a camera to one. They estimated the boundaries of the chessboard both in the Lidar and the camera system in order to establish 3D line correspondences. Although their approach could simultaneously resolve the intrinsic parameters of the camera, the authors specifically focused on reducing the required number of poses for the automatic extrinsic calibration case. The implementation of this method was given as a function as well as an independent GUI application in the Matlab programming platform [15]. Geiger et al. [16] took advantage of multiple non-coplanar chessboards attached to the internal room’s walls to determine the 6-DOF transformations of multiple cameras with respect to a range sensor using a single shot. Apart from the chessboard-based approaches, the category of planar calibration targets has recently been extended by methods that utilize planar boards with patterns of circular holes [11,17]. Whilst such methods enable robust feature extraction between both data representations, they presuppose an elaborate construction of the calibration target by specialized machines, such as CNC.

On the other hand, in the past years, there has been a growing interest in targetless calibration employing scene feature correspondences [18], mutual information among imagery and range data [19], and, more recently, deep learning techniques [20]. Also avoiding the exploitation of a calibration object, Park et al. [21] suggested a two-step (initialization and refinement) approach for spatiotemporal calibration of a camera–Lidar system, taking into consideration the Lidar and camera trajectory deduced by Lidar registration and visual odometry, respectively.

In this paper, we propose a camera–Lidar extrinsic calibration technique introducing a new calibration planar object. The main purpose was to ensure the maximum accuracy and reliability of the method while keeping it as simple and flexible as possible. More specifically, our attempt was based on the following assumptions and objectives concerning the calibration object:

(i) The achievable accuracy of object-based calibration techniques is more controllable by the user compared with that of targetless approaches. An extra capture of the target at a new viewing position may significantly increase the calibration result with practically zero overhead of capturing and processing time.
(ii) The construction of the target should be simple for non-specialized users and should include readily available and low-cost materials. Planar boards are prevalent calibration targets; however, complicated patterns, such as those with circular holes [11,17], require elaborate construction and specialized equipment. Additionally, the size of the proposed target should be kept relatively small compared with the existing calibration approaches of planar targets, allowing a more versatile calibration procedure for outdoor and indoor environments.
(iii) The dimensions of the calibration object are arbitrary (unstructured object) in contrast to most target-based approaches (e.g., [7,11,12,14,17]). In this way, the construction of the target remains as simple as possible, and the calibration results are not affected by any possible manufacturing defect or inaccurate measurement.

Among the different tasks of the extrinsic calibration process, one of the most crucial is the detection of the target in the reference system of all available sensors. Even though in chessboard-based calibration approaches, the calibration object is identified automatically in images and point clouds, there are cases in which this is not always feasible. For instance, the existence of several planar objects in a scene apart from the chessboard plane, a case quite common in interior environments, can lead to misidentification of the chessboard in the range data and degrade the performance and the reliability of the calibration process. Additional difficulties may be introduced in the detection of the chessboard edges in the range data when they have been interrupted by the user’s fingers. In all previous cases, the intervention of the user was mandatory to resolve the issues. Unlike typical chessboard calibration techniques, our approach can overcome many of these limitations (see Section 4), particularly due to the characteristics of the proposed calibration target, without sacrificing the simplicity and effectiveness of the calibration procedure.

Along with geometrical calibration, the user can perform temporal calibration for the synchronization of the camera and the Lidar frames. This is a common issue in systems that lack the capability for hardware synchronization, and it can be performed as a second step after geometrical calibration. Finally, our calibration algorithm is provided as an open-source GUI software implemented in the ROS platform, making it easily accessible to users for research, development, and production purposes. The code is available at https://github.com/up2metric/cam2lidar (accessed on 21 July 2022).

## 2. Methodology

**Figure 1:**
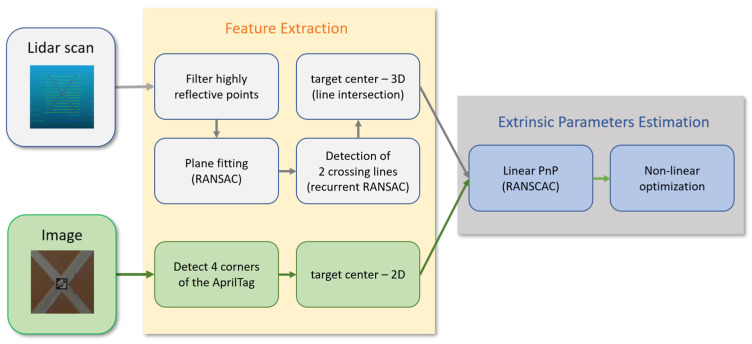
The outline of the geometrical calibration algorithm.

The proposed approach is based on the establishment of dense and highly accurate pairs of corresponding points among Lidar scans and RGB images, aiming at the recovery of the exterior orientation parameters (exterior calibration) of the camera with respect to the Lidar sensor. The internal parameters of the camera were acquired first by exploiting open-source camera calibration toolboxes available in the computer vision community (e.g., OpenCV [22], faucal toolbox [23], and UAV-based calibration [24]). The outline of the geometrical calibration is presented in Figure 1. First, in the *Feature Extraction* phase, 3D to 2D point correspondences are defined among the Lidar and camera reference systems. Using the extracted point pairs, the algorithm estimates the extrinsic parameters of the camera in the Lidar coordinate system.

Subsequently, a methodology for temporal calibration is also presented to synchronize the 3D range recordings of the Lidar sensor to the imagery. Both the geometrical and temporal calibration procedures focus on ease of use and high precision of the computed parameters (i.e., 3D pose and time difference).

### 2.1. Feature Points Extraction

A key component of this endeavor is the exploitation of a calibration object that is easy to set up and ensures the precise detection of corresponding Lidar and camera points. For this reason, a calibration target was deployed that combined the integration of an AprilTag visual fiducial mark [25] along with a Lidar marker. The main concept behind utilizing a single AprilTag is to help define the coordinates of the target center in the image frame with the highest possible accuracy. Thus, and in contrast to other approaches that exploit similar fiducial marks [12,17], our approach avoids estimating the 3D location of the AprilTag in the camera system—a sensitive and error-prone procedure, especially for small marks—and estimates just the center of the AprilTag on the image using the 4 detected corners of the mark on the image frame. The two lines connecting the diagonal corner points of the marker are intersected for the estimation of the center point. Consequently, the size of the marker is irrelevant to the calibration process, and its dimensions can be arbitrarily defined.

**Figure 2:**
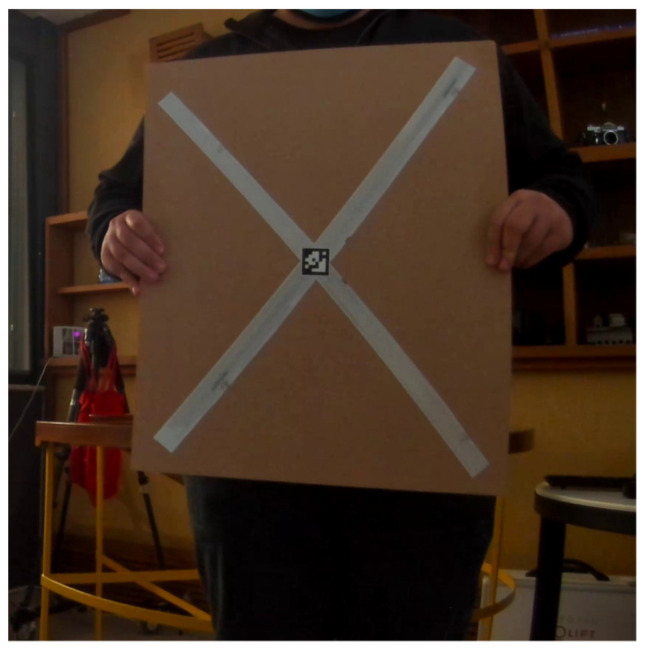
A close view of the proposed calibration object containing two crossing retroreflective stripes and the ApriTag fiducial mark in the middle.

Regarding the Lidar marker, two reflective crossing stripes were attached to a planar board, while at their intersection point, a small AprilTag marker was carefully placed, ensuring the precise alignment of both center points (Figure 2). We kept the size of the AprilTag relatively small (3 cm) to keep the construction of the calibration target as simple as possible. The target center was extracted (a) in the Lidar reference system as the 3D intersection point of the two detected stripes and (b) in the camera reference system as the 2D AprilTag center point.

We proved experimentally that such a small AprilTag size (a few centimeters), considering our 4K camera employed in the calibration procedure, could be automatically detected and precisely localized for a wide range of camera-to-target distances, reaching a maximum distance of 6 m, quite adequate for our sensor configuration.

**Figure 3:**
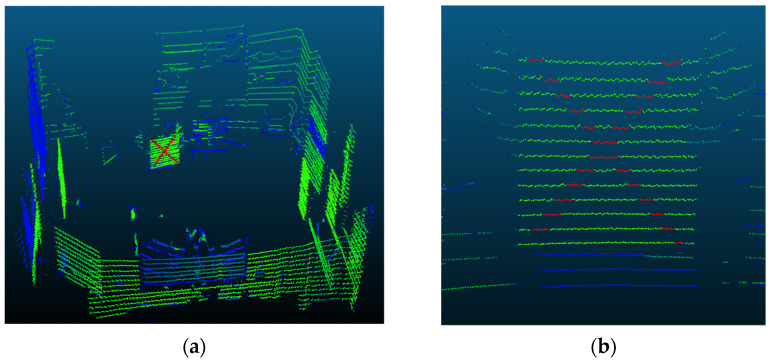
Pseudocolor representation of the laser beam return (intensity value) for a scan generated during the calibration process. (**a**) Overall view of the room. (**b**) View of the calibration target with the two distinct reflective stripes.

Like the image marker, the size of the Lidar marker (planar board and stripes dimensions) is also irrelevant to the calibration procedure. Its center is accurately estimated from the two crossing stripes, provided that each stripe is covered by several successive laser points (min 2–3 points) across and several laser rows (min 3–4 Lidar channels) along its length. In the example in Figure 3, due to the close distance of the camera to the target, the length of the two retroreflective stripes is given by 12 and 13 Lidar channels (we employed Velodyne PUC VLP-16, containing 16 channels), while an average of 9 consecutive Lidar points covers their width. In our calibration examples, the distance between the camera and the Lidar was relatively small (a few centimeters)—a common configuration, especially in mobile mapping systems—allowing the realization of measurements in close ranges from both sensors. With this in mind, we created a relatively small calibration board (45 × 55 cm), shown in Figure 2, which contains two crossing reflective stripes (3 cm wide each) and a small (3 × 3 cm) AprilTag marker aligned with the center of the board, that can yield very satisfactory calibration results (Section 4). For configurations with longer camera-to-Lidar distances, for which calibration measurements are made at large distances from the sensors, a proportionally larger calibration target with a larger AprilTag marker may be utilized for the best performance.

The detection of the Lidar target was carried out automatically due to the distinctiveness of the retroreflective stripes. Setting a minimum threshold (e.g., value 240 in the 8-bit scale) for the recorded intensity of all laser beam returns, an initial clustering was carried out, which effectively eliminates all non-target points (low reflectivity measurements). In Figure 3, the highly reflective points of the calibration target are presented in red, compared with the remaining measurements that correspond to a weaker signal return (green and blue points).

In the second step, the equation of the board plane was estimated using a least-squares minimization approach for all target coplanar points. The employment of only the retroreflective 3D points in the estimation of the board plane equation proved to be more accurate than exploiting all Lidar points on the board (this segmentation procedure is applied in most chessboard-based calibration methods) since the highly reflective materials increased the precision of the range measurements. The target’s 3D points were projected perpendicularly onto the estimated plane and served as the basis for the remaining 2D calculations.

Subsequently, applying the RANSAC [26] method in a sequential manner, a line fitting approach could robustly extract the two distinct crossing lines, given as the middle lines of the two corresponding point groups. The two lines were then intersected to retrieve the 2D location of the target center, which was transferred back to the 3D space by utilizing the estimated plane parameters.

**Figure 4:**
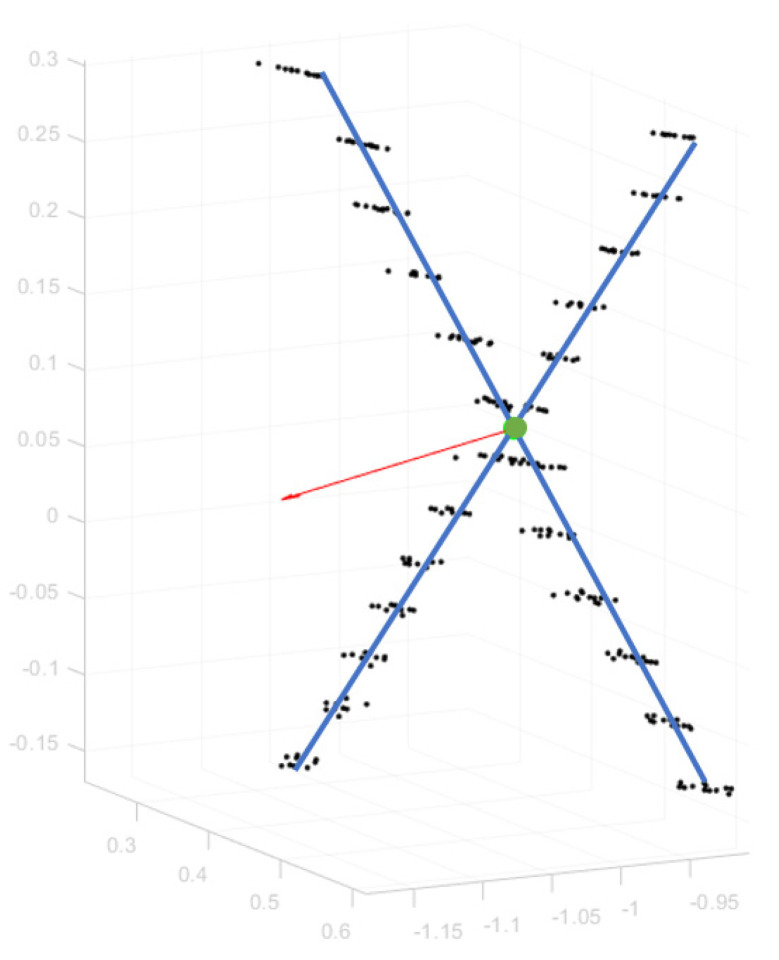
A detailed 3D view of the calibration target with the 3D points corresponding to extracted retroreflective stripes, two detected center lines (in blue), the normal vector of the fitted plane (in red), and the final estimation of the intersection point (in green).

In Figure 4, the highly reflective Lidar points of the target stripes are illustrated, along with the normal vector of the fitted plane (red arrow), the two extracted center lines (blue segments), and the estimated 3D center point target (in green). The severe depth noise of the Lidar measurements, clearly apparent in the figure, along with the significant difference between horizontal and vertical resolution of the laser scanner, were compensated by the plane and line regression stages.

The accuracy of the extracted target center may be improved by merging several (2~3) successive static scans of the target, provided that the target remains stationary during the capturing phase. Using this technique, the precision of the estimated target center may be further increased, yielding a more accurate alignment between the Lidar and camera measurements.

The result of the feature extraction stage was the establishment of accurate 3D−2D point correspondences between the Lidar and the camera reference system from successive measurements of the proposed calibration target from both sensors. These measurements were the inputs of the geometrical and temporal calibration procedures.

### 2.2. Geometrical Calibration

In the preliminary stage, the internal parameters of the camera should be accurately estimated. This procedure can be carried out by utilizing open-source toolboxes, such as the OpenCV camera calibration for ROS [27], the Matlab calibration toolbox [28], or fauccal [23]. Such approaches take advantage of a single and typical planar chessboard, observed by the camera from different orientations and relatively small distances. For longer imaging distances, a multi-chessboard calibration scheme may also be adopted [24].

Utilizing the current feature extraction technique, a sufficient number of point correspondences between 3D Lidar and 2D image points needs to be established for a reliable and accurate exterior calibration. In the first step, the camera exterior orientation is initialized with respect to the Lidar world system by a linear PnP (Perspective-n-Point) [29,30] solution in conjunction with RANSAC to handle outliers.

The 6 estimated extrinsic parameters (3 rotations and 3 translations) are further refined by applying a non-linear least-squares PnP minimization. The cost function that is minimized (Equation (1)) estimates the L2 Norm (Euclidean distance) of the errors between extracted 2D image points and the projected 3D Lidar points in the image (reprojection error):

(1) $$\underset{i}{\sum }\|p_{i}-p\left(K,R_{i},t_{i},P_{i}\right)\|$$

where $p_{i}$ is the target’s center point detected in the image *i*, $p\left(K,R_{i},t_{i},P_{i}\right)$ is the projection of the corresponding Lidar point $P_{i}$ in image *i*, *K* is the matrix containing the camera intrinsic parameters, and *R_i_* and *t_i_* are the rotation matrix and translation vector, respectively [6].

### 2.3. Temporal Calibration

Following the geometrical calibration procedure, a temporal calibration is applied to address synchronization issues between the camera and Lidar recordings. This is a severe problem that occurs, especially in camera–Lidar systems that lack hardware synchronization (i.e., non-synchronized triggering or irrelevant timestamps).

The algorithm described here attempts to match the 2D location of an extracted AprilTag fiducial from a specific image frame to the closest 2D image location of several successive Lidar target points after being projected onto the specific image. Let $p_{i}$ be the 2D center of the calibration target detected on image *i* (AprilTag marker) and $P_{i}$ be the corresponding (according to the non-synchronized timestamps) 3D point extracted from the Lidar data using the intersection of the two retroreflective stripes. Having recovered the rigid transformation between the camera and the Lidar from a preliminary geometrical calibration stage, the projection of point $P_{i}$ onto image *i* should coincide (within the limits of the calibration error) with image point $p_{i}.\;$Nevertheless, due to the time-shifting between the camera and Lidar recordings, the two image points are separated by an image distance proportional to the unknown time shift as well as to the velocity of the moving calibration target. By sequentially projecting on image *i* all 3D target points $P_{i-t/2}$ to $P_{i+t/2}$ that have been extracted from different close-in-time measurements of a constantly moving target (giving a time interval *t*), the temporal calibration algorithm selects the projection $p_{j}$ of that point with the minimum distance in pixels from the reference image point $p_{i}$ and assigns the timestamp of the selected Lidar frame *j* to the current image frame *i*.

This implies that the calibration target should be moving at a relatively constant speed during data capturing to prevent possible temporal misalignment. Apart from the constant velocity of successive target detections (target tracking), another criterion is enforced here that concerns the direction of the target’s movement. According to this, an angular threshold bounds the direction of every new detected target within an acceptable angular range with respect to the direction of the preceded target. Thus, the possibility of mismatching images to Lidar targets is further reduced.

Combining several such target measurements from different times during the acquisition, the algorithm can accurately infer the overall time-shift of the camera with respect to the Lidar recording.

## 3. Calibration Workflow

Our calibration algorithm is provided as an open-source implementation in the ROS framework and is primarily aimed at non-expert users by keeping the calibration approach as simple and effective as possible. In addition, the high accuracy achieved in locating the target in both sensing systems (camera and Lidar) can establish the proposed algorithm as a key calibration tool, not only for research but also for production purposes, allowing it to be also used during the evaluation similar calibration approaches (see Section 4).

The ROS [30] platform was chosen due to its great acceptance among the scientific community of robotics. The ROS is an open-source robotic framework that aims to resolve the main challenges that robotics engineers have to cope with concerning perception, navigation, and manufacturing tasks and support them in the many stages of the development and production phases.

For our calibration toolbox, the input data should be compatible with the requirements of the ROS framework. Two different topics are required: one for imagery containing camera messages (uncompressed image data) and one for 3D scans containing messages from the Lidar (point cloud messages). The internal parameters of the camera are fed to the application using a camera info topic or as an external file containing a camera matrix and distortion coefficients. It is also possible for the user to store the topics in a rosbag file and perform the calibration offline.

**Figure 5:**
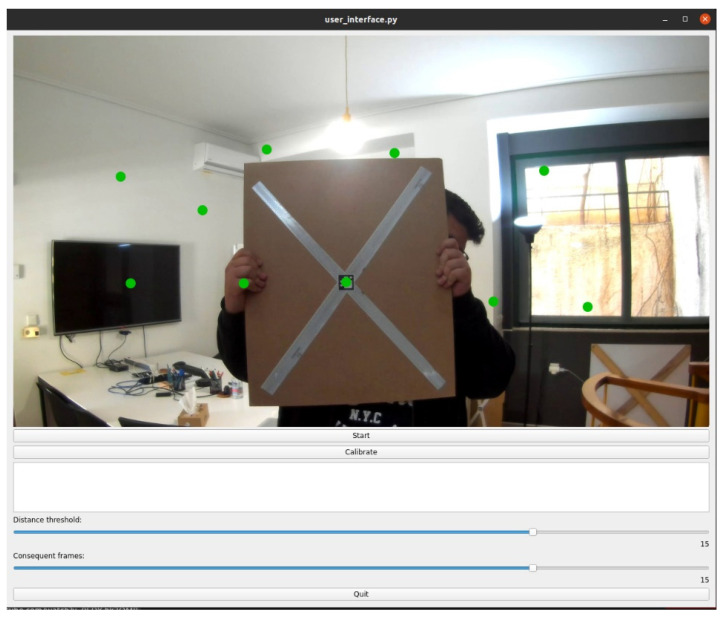
The graphical user interface of the proposed calibration application displaying the feed from the camera and two sliders for setting the parameters “distance threshold” and “consequent frames”. The image displays the positions of the current and previously accepted measurements.

Regarding geometrical calibration, first, the software takes successive target measurements while the user moves the calibration target to various places. For increased calibration accuracy, the ‘active area’ corresponding to the overlapping FOV of both sensors ought to be covered by an adequate number of equally spaced target measurements. To this end, the algorithm controls the target’s density by considering only measurements that satisfy the minimum distance criterion to each other. Moreover, from the displayed locations of all previous measurements (Figure 5), the user can decide the next target positions.

Aiming to achieve reliable results, even in configurations with significant time delays among camera and Lidar recordings, we utilized an image tracking method to eliminate abrupt target movements. Thus, the algorithm can detect slow-moving sequences of the target in real-time and take a measurement only when the target is practically stationary. This is achieved by the parameter “distance threshold”, which concerns the minimum displacement from frame to frame, and parameter “consequent frames”, which determine the maximum number of consequent frames being taken into consideration (Figure 5). This capability can significantly affect the quality of the calibration process, especially in non-synchronized recordings.

**Figure 6:**
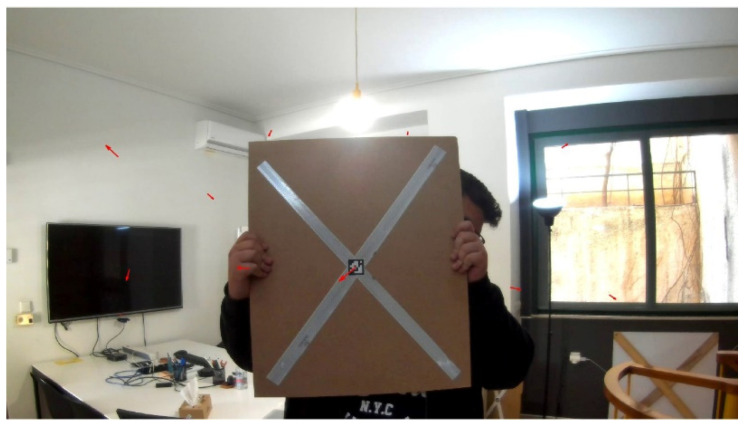
The reprojection errors are given as directional vectors for all measurements involved.

After the capturing phase, the users can visualize the calibration results in the command window and the spatial distribution of the residual (reprojection) errors displayed on the image for every observation individually. In Figure 6, the residual errors of several observations are given in one of the calibration frames as vectors with their corresponding length and direction on a scale 10 times larger than the real values.

In addition to the geometrical calibration, the user, exploiting a similar interface, can perform temporal calibration in order to synchronize the camera to the Lidar frames. In this case, and in contrast to the previous approach, the distance parameter is applied here as a minimum ‘distance threshold’ among consecutive target observations, which aims to prevent the measurement of slow-moving targets. A target observation is considered valid only if the minimum distance criterion is met for an adequate number of concurrent frames (the ‘consequent frames’ parameter).

Since the geometrical calibration is based on the accurate synchronization of the recordings captured by the camera and the Lidar sensor, it is advisable, especially for datasets with larger synchronization differences, to repeat the geometrical calibration after the synchronization step.

## 4. Evaluation and Further Results

**Figure 7:**
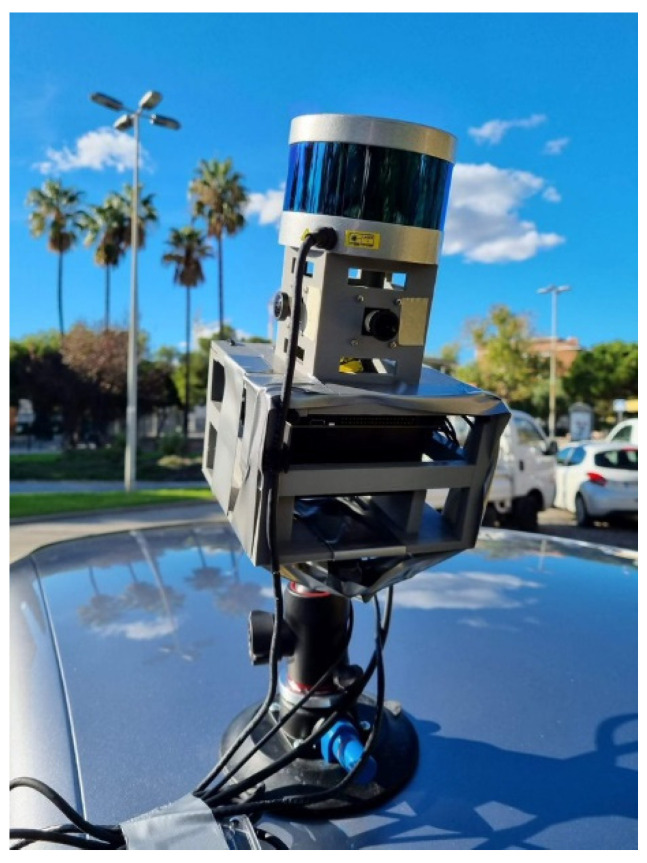
The mobile mapping system (MMS) mounted on top of a car.

In this section, we present experimental results to evaluate the accuracy of our calibration methodology as well as the effectiveness and flexibility of the proposed ROS implementation. Our software was exploited for the calibration of a custom-made mobile mapping platform consisting of four embedded 13MP machine vision cameras (by e-con Systems) with respect to a Velodyne PUC VLP-16 Lidar sensor mounted on top of the cameras (Figure 7). A thorough description of the current mobile mapping platform is given in [3].

Since the current configuration of the MMS does not allow any image overlap between the four cameras, the procedure is mainly based on the independent calibration of each camera with reference to the Lidar sensor. First, the interior orientation of each camera was estimated using fauccal [23]. After accurately recovering the camera’s internal parameters, the rigid body transformation between the Lidar and the camera coordinate systems can be accurately recovered using the studied approach. An adequate number (average 12 points) of well-distributed observations of the calibration target was measured, evenly covering the overlapping area of every camera with the Lidar sensor. Since the distance of each camera from the Lidar was small (7~8 cm) in the current setup, the large overlapping area allowed measurements to be taken at close ranges (2–6 m) to the sensors. Therefore, the calibration procedure was performed indoors. A small calibration target was also utilized for the same reason (see dimensions in Section 2.1), making the procedure more flexible and faster. For larger camera–Lidar configurations, the calibration should be performed outdoor to cope with the narrow overlap of the two FOVs at the cost of using a relatively larger calibration target. The computed exterior orientation parameters of all cameras along with the reprojection error of the adjustments can be found in Table 1.

**Figure 8:**
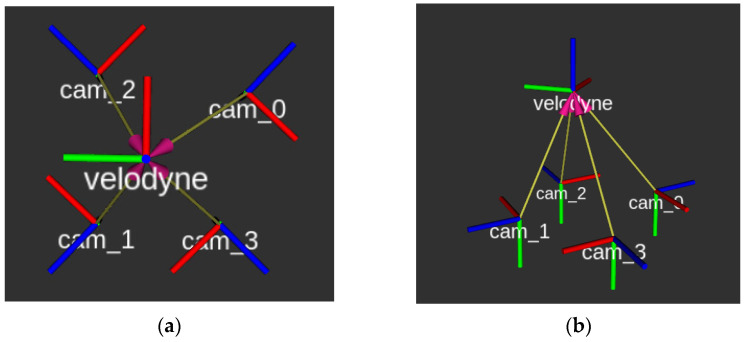
A graphical representation of the transformation of the four camera systems (cam_0, cam_1, cam_2, and cam_3) with respect to the Lidar system (Velodyne). (**a**) Top view. (**b**) Perspective view.

The results are quite satisfactory given primarily the modest pixel error of the adjusted observations. It was also observed that the parameters of the four camera-to-Lidar transformations were noticeably consistent and apparently close to the real relative positions and rotations of the sensors. This fact also holds in Figure 8, in which the coordinate systems of the four machine vision cameras are visually represented with respect to the Lidar reference system. It was proven that the cameras were positioned symmetrically with respect to the Lidar sensor.

**Figure 9:**
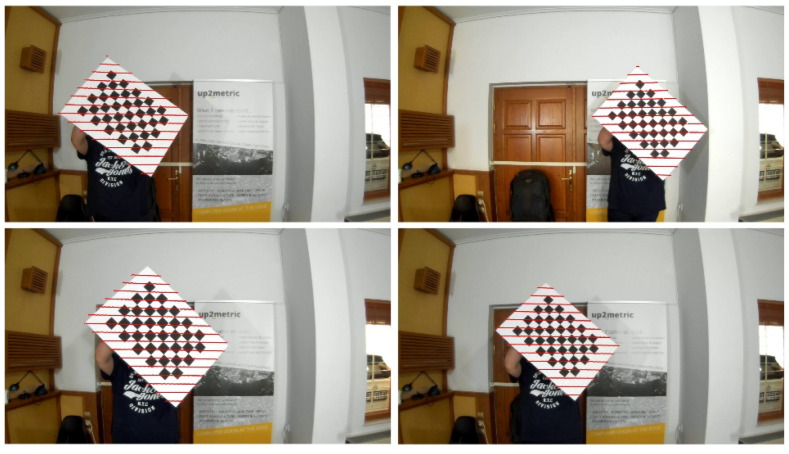
The Lidar points corresponding to the chessboard are displayed on four calibration frames using the estimated extrinsic calibration from Zhou’s method.

For the purposes of comparison, the developed calibration approach was also evaluated against the results from a rigorous open-source calibration toolbox commonly adopted by the mobile mapping community [14]. Eleven images of a chessboard plate with different orientations were captured by the camera (cam_0), while the scene was scanned simultaneously by the Velodyne Lidar sensor. According to Zhou et al.’s method, the common observations of the 3D chessboard edges extracted automatically in both the camera and the Lidar system allow the accurate estimation of the external parameters. The calibration was performed following the Matlab implementation of this method [15]. In Figure 9, four such frames of the chessboard are presented, along with the corresponding Lidar points projected on top (point stripes in red) and after the determination of the camera’s exterior orientation.

For a more reliable assessment of both calibration results, a set of well-defined 3D points was projected onto the camera frame using the external parameters yielded by both solutions independently (our method and Zhou’s method). The 3D points were extracted by moving our proposed calibration target into different positions while it was being recorded by the camera and the Lidar device. The 3D target centers extracted from the Lidar scans were projected onto the image and compared against the corresponding 2D target centers extracted on the image, giving the final RMSE for both datasets.

The deviations and the computed RMSE of the 22 measurements for the two approaches are shown in Table 2. It is worth mentioning that the 22 measurements did not participate in our calibration solution so as to keep the final results unbiased. The achieved accuracy of both methods was considered satisfactory, with our approach performing slightly better.

However, the difference between the two is considerably greater if one considers the convenience of the calibration process. In the case of Zhou et al.’s method, and according to our experience with the corresponding Matlab 2022a implementation, there are a lot of prerequisites one should consider for reliable calibration. First, the scans of the chessboard plate should not be disturbed by any obstacle, particularly the user’s fingers holding the plate, which is a very common case. Similarly, the user should not keep the plate too close to his body. Both cases can significantly deteriorate the calibration results by intervening either during the edge extraction of the 3D chessboard (the former case) or in the determination of the planar board equation (the latter case). Our approach can directly overcome these barriers, keeping only the structural information of the reflective target points. Additionally, and apart from the requirement for tilted checkerboards during the acquisition phase, the checkerboard-based algorithm did not perform satisfactorily when detecting the board planes from the successive scans, especially in indoor environments with many planar objects present. In several 3D scans, user interaction was inevitable. On the other hand, the proposed calibration technique does not require any a priori structural information for the calibration target, such as checkerboard square size or padding dimensions, keeping the workflow as intuitive and flexible as possible for the user. The only requirement is the creation of the current calibration object with two reflective stripes in a crossing layout, which is a rather simple and low-cost endeavor.

**Figure 10:**
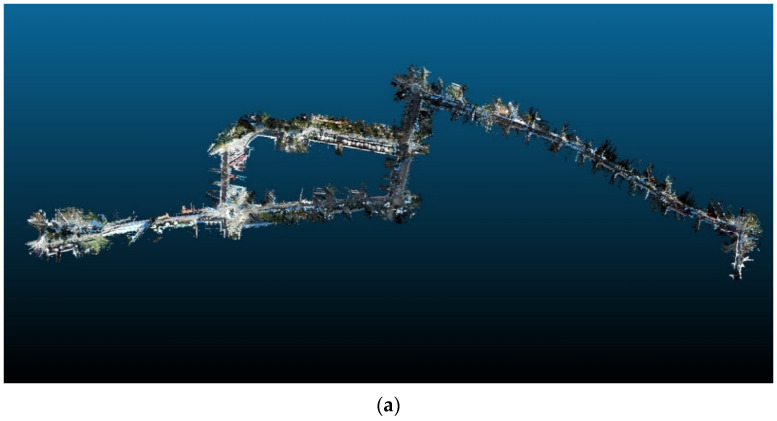

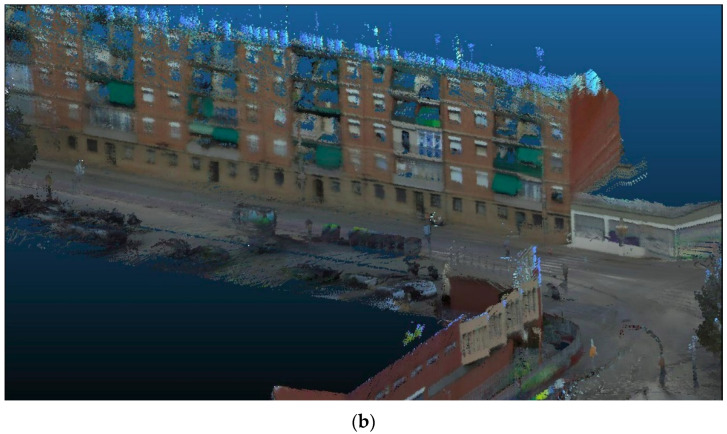
The point cloud generated with the mobile mapping system after texture-mapping with our custom algorithm, exploiting the estimated calibration parameters. (**a**) Top overall view. (**b**) A detailed view.

Finally, the calibrated relative rotations and translations between the four cameras (camera rig) and the Lidar sensor of our mobile mapping system were assessed during a SLAM and a texture mapping procedure. A final 3D map was generated employing the Lidar-based SLAM approach of the RTAB-Map package [31] from 10,981 successive Velodyne scans for a 3.8 km trajectory in the city center of Barcelona. Our custom implementation of a multi-image texture-mapping approach successfully colorized the generated 3D map consisting of approximately 50,000,000 points by combining color information from multiple images while exploiting different visual and geometrical criteria. Further information on this approach will be given in an upcoming publication. Figure 10 demonstrates the high visual quality of the produced texture-mapped model after recovering the external calibration parameters of the MMS using our calibration software.

## 5. Conclusions

In this contribution, we present a novel approach for automatic camera-to-Lidar extrinsic and temporal calibration based on an easy-to-create calibration target. For the geometrical (extrinsic) calibration, the 3D rigid transformation of the camera system is estimated with respect to the Lidar frame on the basis of the establishment of 2D to 3D point correspondences. The temporal calibration (sensor synchronization) is carried out by matching the position of the calibration target detected on the image frame to the corresponding 3D target projected on the same. The geometrical calibration was evaluated against a state-of-the-art approach, and it yielded very satisfactory results. Both geometrical and temporal calibration can be carried out by the user through our open-source implementation in ROS. The effectiveness and reliability of the current approach make this toolbox suitable for production purposes and for researchers during the evaluation of their own calibration algorithms. In the future, we plan to integrate a more rigorous model for the plane-fitting step and to further evaluate the proposed calibration method with measurements from various cameras and Lidar sensors. Finally, we intend to make our toolbox available for Matlab users.

## Figures and Tables

**Table 1:** Calibration results for all four cameras of a mobile mapping system.

Camera Id	cam_0	cam_1	cam_2	cam_3
σ_ο_ (pixel)	3.7	4.4	3.2	4.1
ΔΧ (cm)	3.7	−3.3	3.4	−2.8
ΔΥ (cm)	−3.5	3.6	2.7	−2.3
ΔΖ (cm)	−7.0	−7.4	−7.2	−7.3
omega (deg)	179.70	1.14	−0.52	−179.92
phi (deg)	46.81	−48.19	43.77	−44.05
kappa (deg)	89.94	−88.50	−89.32	89.38

**Table 2:** Comparison results between Zhou’s method and our approach for a dataset of 22 checkpoints.

	Zhou Appr.	Our Appr.
Num of checkpoints	22	22
RMSE_x (pixel)	14.2	6.3
RMSE_y (pixel)	16.4	9.6

## Data Availability

The data presented in this study are available on request from the corresponding author.
